# The Prevalence of Fibromyalgia in Rheumatoid Arthritis Patients Using the Fibromyalgia Assessment Screening Tool (FAST 4) Based on the Multidimensional Health Assessment Questionnaire (MDHAQ)

**DOI:** 10.7759/cureus.64011

**Published:** 2024-07-07

**Authors:** Hind El-Kasmi, Bouchra Amine, Anbar Kabbaj, Samira Rostom, Imane El Binoune, Samia El Hilali, Rachid Bahiri

**Affiliations:** 1 Rheumatology A, El Ayachi Hospital, Ibn Sina University Hospital, Rabat-Salé, MAR; 2 Laboratory of Community Health (Public Health, Preventive Medicine and Hygiene) Department of Public Health, Faculty of Medicine and Pharmacy, Mohammed V University, Rabat, MAR; 3 Laboratory of Biostatistic, Clinical and Epidemiological Research, Department of Public Health, Faculty of Medicine and Pharmacy, Mohammed V University, Rabat, MAR

**Keywords:** first, fast, fibromyalgia rapid screening tool, multidimensional health assessment questionnaire, disease activity score 28 joint count, fibromyalgia assessment screening tool, rheumatoid arthritis, fibromyalgia

## Abstract

Introduction

Fibromyalgia (FM) is characterized by widespread pain and fatigue, accompanied by symptoms such as decreased concentration, autonomic dysfunction, and abdominal pain. It can be either primary or secondary, notably to rheumatoid arthritis (RA). The Fibromyalgia Assessment Screening Tools (FAST 4), derived from the Multidimensional Health Assessment Questionnaire (MDHAQ), is a composite tool allowing for the rapid screening of FM. Our primary objective is to determine the prevalence of FM among RA patients using the FAST 4 index. Secondary objectives include comparing the FAST 4 index with the FiRST score and describing the correlation between FM and RA activity and different factors associated with FM in RA patients.

Methods

This was an observational cross-sectional study including patients diagnosed with RA according to the ACR/EULAR criteria. The FAST questionnaire comprises four sections assessing pain and fatigue on a visual analog scale, painful joints reported by the patient, and a list of 60 symptoms. A FAST 4 score of ≥ 3/4 indicates a positive screening for FM. Demographics and disease features were compared using descriptive statistics. Univariate and multivariate analyses using logistic regression models were performed to calculate odds ratios (ORs) with 95% CI. The sensitivity and specificity of the FAST 4 index were evaluated, and Fagan’s nomograms were used to illustrate post-test probability. Statistically significant results were considered for p-values less than 0.05.

Results

The study enrolled 97 patients diagnosed with RA. The mean age of the patients was 56 ± 12.7 years, with a predominance of females (90.7%, N=88). The mean duration of RA was 13.5 ± 8.69 years. RA activity measured by DAS 28-ESR showed that 40.2% (N=39) had high disease activity, 38.1% (N=37) had moderate disease activity, 11.3% (N=11) had low disease activity, and 10.3% (N=10) were in remission. The prevalence of comorbid FM, according to the FAST 4 index, was 30.9% (N=30). Based on the Multidimensional Health Assessment Questionnaire (MDHAQ), depression was observed in 66.7% (N=20) patients with FM, while anxiety was reported in 60% (N=18). Moreover, 30.4% of patients screened positive for FM using the FiRST score. The FAST 4 index detected FM patients defined by FiRST with a sensitivity of 78.6% and a specificity of 87.1%. The positive predictive value (PPV) was 73.3%, and the negative predictive value (NPV) was 90%.

Univariate analysis revealed that a positive FAST 4 index was associated with the number of painful and swollen joints (p<0.001 and 0.03, respectively). Additionally, patients with a positive FAST 4 index showed higher DAS 28 scores (p=0.002). No significant association was found with CRP levels (p=0.328), ESR (p=0.499), or the use of biological treatments (p=0.146) or corticosteroids (p=0.940). In multivariate analysis, only depression remained a risk factor, increasing the risk sixfold with an OR of 5.917, 95% CI (1.91-18.3), p=0.002.

Conclusion

Our study suggests a high prevalence of concomitant FM in our population, highlighting the importance of screening for FM, particularly using the FAST 4 index based solely on the MDHAQ questionnaire.

## Introduction

Fibromyalgia (FM) is characterized by widespread pain and fatigue, accompanied by symptoms such as decreased concentration, autonomic dysfunction, and abdominal pain [1]. Research suggests that abnormal central pain processing and central sensitization are key mechanisms involved. Classified under central sensitivity syndromes, FM often coexists with chronic fatigue syndrome, irritable bowel syndrome, and myofascial pain [2].

FM can manifest as a primary condition or as a secondary complication related to another existing health disorder. Studies have reported FM in approximately 20-30% of patients with rheumatic diseases and 5%-52% of patients with rheumatoid arthritis (RA) [3,4]. Comorbid FM may lead to an overestimation of disease activity, influencing treatment responses and potentially leading to unnecessary escalation of therapy [5].

Despite its prevalence, no gold standard for diagnosis exists. In 2011, the American College of Rheumatology (ACR) updated the diagnostic criteria established in 1990, incorporating the patient self-report pain index and symptom severity scale questionnaire [6,7]. However, its applicability in routine practice is challenging. Consequently, several scores have been developed for the rapid screening of FM, including the Fibromyalgia Rapid Screening Tool (FiRST), which is a screening score used to detect FM in less than 3 minutes, and the FibroDetect® [8,9].

The Multidimensional Health Assessment Questionnaire (MDHAQ) is a patient self-report tool adapted from the Health Assessment Questionnaire (HAQ), widely used in routine clinical care. The MDHAQ, which can be completed in 5-10 minutes, has proven useful in assessing various rheumatic diseases [10]. The Fibromyalgia Assessment Screening Tool (FAST 4) is a practical cumulative index using MDHAQ to screen for FM in patients with rheumatic diseases [7,11]. It includes a cumulative score of the symptom checklist, painful joint count, and both pain and fatigue visual analog scales (VAS). A FAST 4 score ≥ 3 correctly classified 91.7% of patients compared to the 2011 FM criteria, with a sensitivity of 70.4% and a specificity of 97.1% [7].

In this study, our primary objective is to determine the prevalence of FM among RA patients using the FAST 4 index. Secondary objectives include comparing the FAST 4 index and the FiRST score and describing the correlation between FM and RA activity and different factors associated with FM in RA patients.

## Materials and methods

Patient selection and study design

We conducted an observational cross-sectional study at a single university hospital center, including all patients diagnosed with RA who met the American College of Rheumatology/European League Against Rheumatism (ACR/EULAR) criteria [12] and who were older than 18 years. We adhered to the Strengthening the Reporting of Observational Studies in Epidemiology (STROBE) cross-sectional checklist while preparing our report [13].

Patient questionnaire

The Multidimensional Health Assessment Questionnaire (MDHAQ) includes a self-reported painful joint count, modified from the rheumatoid arthritis disease activity index (RADAI), which asks patients to score pain in 16 specific joint groups, eight on each side. The scoring options are 0 (no pain), 1 (mild pain), 2 (moderate pain), or 3 (severe pain). The modified version also includes the neck and back, resulting in 18 joints scored from 0 to 3, yielding a total score range of 0 to 54 [14]. Additionally, a 60-symptom checklist, which includes "Yes/No" responses to common symptoms, is designed to screen for early detection of disease flares, comorbidities, and adverse effects of medication. The MDHAQ incorporates a pain and fatigue VAS. The FAST 4 index is a composite cumulative index where one point is assigned for each of the following: pain VAS ≥ 6, fatigue VAS ≥ 6, RADAI ≥ 16/54, and 60 symptoms ≥ 16/60. A FAST4 score of ≥ 3/4 indicates a positive FM screen [7,15].

Data sources and assessment

Demographic and clinical data of individual patients were collected, including age, gender, profession, body mass index (BMI), comorbidities, duration of RA, and medical and RA disease history. Blood samples were collected during the visit, where the FAST4 questionnaire was completed for measurement of inflammatory markers such as C-reactive protein (CRP) and erythrocyte sedimentation rate (ESR). Additional parameters were retrieved from patients' medical records, including serum levels of anti-cyclic citrullinated peptide (anti-CCP) antibodies measured by enzyme-linked immunosorbent assay (ELISA) and rheumatoid factor (RF). Disease activity in RA patients was assessed using the Disease Activity Score for 28 joints with ESR (DAS28-ESR) and the Health Assessment Questionnaire (HAQ) score for functional assessment.

Ethics approval and consent to participate

The study protocol was reviewed and approved by local institutional review boards and the national ethics committee: the Ethics Committee for Biomedical Research at Mohammed V University in Rabat, Faculty of Medicine and Pharmacy of Rabat. The committee's reference number is CERB 61-24. Written informed consent for publication was obtained from the patients.

Sampling and statistical analysis

The sample size calculation was based on the prevalence estimation formula [16], with an expected prevalence of 10% as reported in the literature, a type I error rate of 5%, and aiming for a precision of 6%. The required sample size for our study was approximately 97 subjects.

Statistical analysis was conducted using JAMOVI version 1.6. Parameters with normal distribution were presented as mean ± standard deviation (SD), while skewed parameters were expressed as median ± interquartile range (IQR, defined as the 25th to 75th percentiles). Qualitative data were presented as frequencies (number and percentage). Comparisons of continuous variables were conducted using Student’s t-test, while categorical variables were analyzed using the Chi-square test or Fisher’s exact test, as appropriate. The sensitivity and specificity of the FAST4 index were evaluated, and Fagan’s nomograms were used to illustrate post-test probability. Furthermore, multivariate analysis was performed using binary logistic regression to examine the interaction of multiple variables in predicting outcomes. In all statistical analyses, a two-tailed p-value of less than 0.05 was considered statistically significant.

## Results

The study enrolled 97 patients diagnosed with rheumatoid arthritis (RA). The mean age of the patients was 56 ± 12.7 years, with a predominance of females (90.7%, N=88). The majority of patients, 86.5% (N=83) were unemployed. Among them, 30.9% (N=30) had hypertension, and 24% (N=23) had diabetes. Additionally, 33.3% (N=25) were overweight, and 34.6% (N=26) were obese. The mean duration of RA was 13.5 ± 8.69 years. Seropositivity for RA was observed in 89.7% (N=87) of patients, with 86.8% (N=79) having erosive RA. The mean CRP level was 7.4 ± 18.3, and ESR was 32 ± 24.7. In terms of RA activity measured by DAS 28-ESR, 40.2% (N=39) had high disease activity, 38.1% (N=37) had moderate disease activity, 11.3% (N=11) had low disease activity, and 10.3% (N=10) were in remission. 70.1% were receiving biological treatment and 68.4% were being treated with corticosteroids. All demographic and clinical characteristics of the patients are summarized in Table 1.

**Table 1:** Baseline characteristics of participants (*): Expressed as mean and standard deviation or median and interquartile range (IQR). BMI: body mass index; CRP: C-reactive protein; ESR: Erythrocyte sedimentation rate; DAS28: Disease activity score for 28 joints; CCP: Cyclic citrullinated peptide; HAQ score: Health assessment questionnaire score; DMARDs: Disease-modifying antirheumatic drug. Data on bone erosions and glucocorticoid use and dose were missing in six and two patients, respectively.

Characteristics	All patients
Patients. N (%)	97 (100)
Age, Years *	56 ± 12.7
Females n (%)	88 (90.7)
Unemployed n (%)	83 (86.5)
Overweight (BMI 25 to 29.9) n (%)	25 (33.3)
Obesity (BMI 30 to 39.9) n (%)	26 (34.6)
Hypertension n (%)	30 (30.9)
Diabetes n (%)	23 (24)
Osteoporosis n (%)	49 (57.6)
Rheumatoid arthritis duration. Years*	13.5± 8.69
Deforming rheumatoid arthritis n (%)	47 (48.5)
CRP (mg/L) *	7.4 ± 18.3
ESR (mm/hr) *	32±24.7
Remission (DAS 28-ESR≤2.6) n (%)	10 (10.3)
Low disease activity (2.6)	11 (11.3)
Moderate disease activity 3.2	37 (38.1)
High disease activity (DAS 28-ESR< 5.1) n (%)	39 (40.2)
Rheumatoid factor (UI/mL) *	135± 351
Anti-CCP (UI/mL) *	103 ± 203
HAQ score*	1.12± 3.8
Bone erosions n (%)	79 (86.8)
Conventional synthetic dmards use n (%)	66 (68)
Methotrexate n (%)	29 (43.9)
Biologic DMARD use n (%)	68 (70.1)
Rituximab n (%)	46 (66.7)
Anti-TNF n (%)	15 (21.7)
Anti-IL6 n (%)	8 (11.6)
Glucocorticoid use n (%)	65 (68.4)

**Figure 1:**
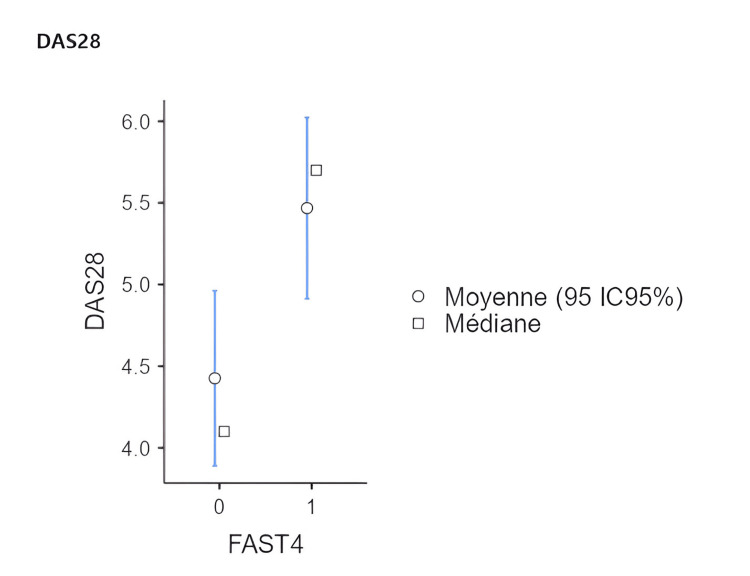
Correlation between positive FAST 4 index and DAS 28-ESR FAST: Fibromyalgia assessment screening tool; DAS 28: Disease activity score for 28 joints

Univariate analysis revealed that a positive FAST 4 index was associated with the number of painful and swollen joints (p<0.001 and 0.03, respectively). Additionally, patients with a positive FAST 4 index showed higher DAS 28 scores (p=0.002) with OR: 1.327, 95% CI (1.012-1.738) (Table 2, Figure 1), as well as the presence of depression and anxiety in the MDHAQ questionnaire (p<0.001 and 0.007 respectively) (Table 3). No significant association was found with CRP levels (p=0.328), ESR (p=0.499), or the use of biological treatments (p=0.146) or corticosteroids (p=0.940) (Table 2). In multivariate analysis, only depression remained a risk factor, increasing the risk sixfold with an OR of 5.917, 95% CI (1.91-18.3), p=0.002.

**Table 2:** Univariate analysis of population with and without FM (*): Expressed as mean and standard deviation or median and interquartile range (IQR). FM: Fibromyalgia; FAST: Fibromyalgia assessment screening tool; CRP: C-reactive protein; ESR: Erythrocyte sedimentation rate; DAS28: Disease activity score for 28 joints; HAQ score: Health assessment questionnaire score; DMARDs: disease-modifying antirheumatic drug. Data on bone erosions and glucocorticoid use and dose were missing in six and two patients, respectively.

Characteristics	FAST 4 (+) n=30	FAST 4 (-) N=67	P-value
Age, years*	58.2± 14.4	55.7± 11.9	0.371
Females n (%)	29 (33)	59 (67)	0.26
Rheumatoid arthritis duration, years*	12± 7.971	14.50± 8.98	0.634
CRP mg/L*	7.5± 19.79	7.25± 17.82	0.328
ESR mm/hr*	35 ±23.488	30± 25.19	0.499
Tender Joint Count (TJC)*	9.0 ±8.435	3.0± 5.92	<0.001
Swollen Joint Count (SJC)*	5.5 ±4.728	3 ±4.22	0.03
DAS28-ESR*	5.70 ±1.470	4.09± 2.17	0.002
Seropositivity n (%)	26 (29.9)	61 (70.1)	0.498
HAQ score*	1.2± 0.527	1.1± 4.35	0.814
Bone erosions n (%)	25 (31.6)	54 (68.4)	1.000
Conventional synthetic DMARDs use n (%)	23 (34.8)	43 (65.2)	0.223
Biologic DMARD use n (%)	18 (26.5)	50 (73.5)	0.146
Glucocorticoid use n (%)	20 (31.1)	45 (69.2)	0.940
Glucocorticoid dose*	5.00± 2.49	5.00 ± 4.04	0.108
Depression n (%)	20 (51.3)	19 (48.7)	<0.001
Anxiety n (%)	18 (46.2)	21 (53.8)	0.007

**Table 3:** Parameters of FAST 4 index, FiRST score, depression, and anxiety in the study population Data are n (%). FAST: Fibromyalgia assessment screening tool; FiRST: Fibromyalgia rapid screening tool; RADAI: A self‐administered rheumatoid arthritis disease activity index; VNS: Visual analogue scale

Parameters	Patients N=97 n (%)
Positive FAST 4 score	30 (30.9)
Pain VNS≥6	51 (52.6)
Fatigue VNS≥6	53 (54.6)
RADAI≥16	30 (30.9)
60-symptom checklist≥16	20 (20.6)
Positive FiRST score	14 (30.4)
Depression	20 (66.7)
Anxiety	18 (60)

**Figure 2:**
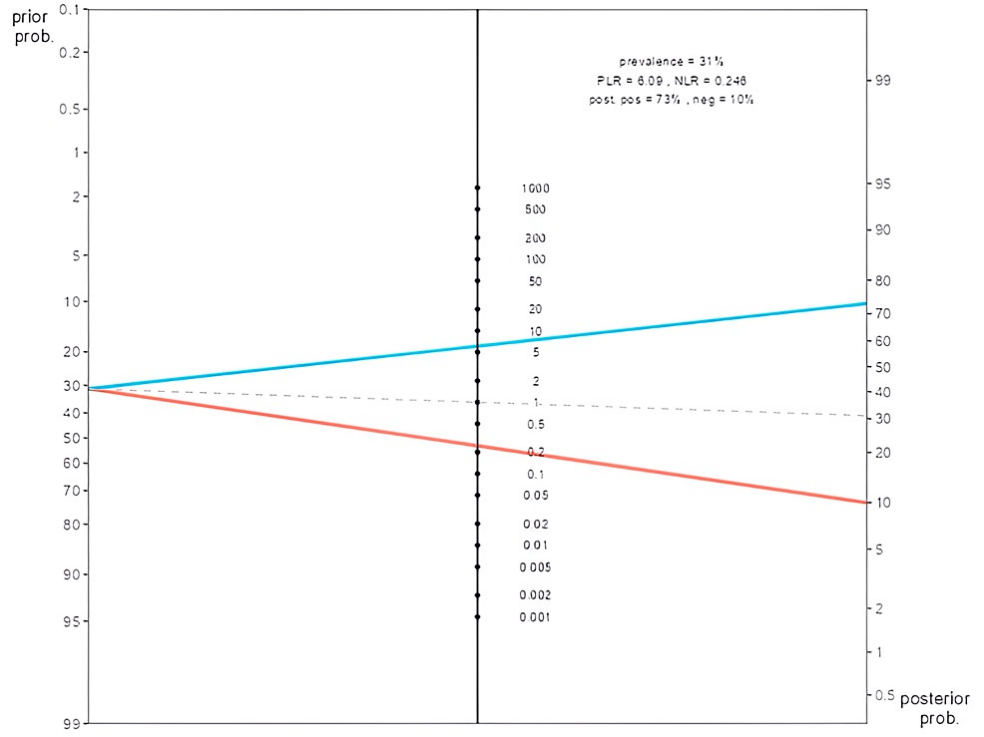
Fagan nomogram to calculate the post-test probability indicates the diagnostic gain The pretest probability corresponds to the prevalence of fibromyalgia found in the study. The middle line represents the values of the likelihood ratios (LR+ and LR-). The post-test probability indicates the diagnostic gain.

The FAST 4 index detected FM patients defined by FiRST with a sensitivity of 78.6%, and the specificity was 87.1% (Table 4). The positive predictive value (PPV) was 73.3%, and the negative predictive value (NPV) was 90%. The positive likelihood ratio (LR+) was six, and the negative likelihood ratio (LR-) was 0.246. This result was illustrated by the Fagan diagram, showing that if the FAST 4 is positive, the probability of having FM in RA patients increases from 31% to 73%. Conversely, if the FAST 4 index is negative, the probability of having FM in RA patients decreases (from 31% to 10%) (Figure 2).

**Table 4:** Sensitivity and specificity of FAST4 in identifying FM in RA patients FAST: Fibromyalgia assessment screening tool. FM: Fibromyalgia. RA: Rheumatoid arthritis.

Diagnostic measure	Value (%)	95% confidence interval
Sensitivity	78.6	49.2-95.3
Specificity	87.1	70.2-96.4

## Discussion

Our study revealed a prevalence of fibromyalgia (FM) at 30.9%. The sensitivity analysis demonstrated comparable performance for the FAST 4 and FiRST scores. The univariate analysis revealed an association between FM and high disease activity, as well as the presence of depression and anxiety, as assessed by the MDHAQ questionnaire. The FAST 4 index detected FM patients defined by FiRST with acceptable sensitivity (78.6%) and good specificity (87.1%).

Chronic inflammation may mediate the transition from peripheral to central pain, resulting in FM symptoms [5]. The reported prevalence of fibromyalgia in RA varies considerably, ranging from 5% to 52% [4]. A cross-sectional longitudinal study including 636 patients in the Oslo RA register found a prevalence of FM at 30%, similar to our findings, using the ACR 2011 criteria [1]. Several factors are associated with increased FM frequency in inflammatory rheumatic diseases, including female gender [17], lower educational status [18], and higher disease activity [4,19-22]. The high prevalence rate in our study might be influenced by the demographic characteristics of our cohort, particularly the high percentage of females (90.7%) and unemployed patients (86.5%), as well as patients with high disease activity according to the DAS28 score (41.1%).

Moreover, the prevalence of FM is influenced by the classification criteria applied. For instance, an Australian study reported a higher prevalence of FM using the 2010/2011 criteria compared to the 1990 criteria (41.9% and 33.3%, respectively) [23]. The FiRST score, consisting of six items related to FM features like pain, fatigue, and sleep disturbances, showed a sensitivity of 90.5% and a specificity of 85.7% in detecting FM [8]. A study of the performance of the FiRST score for detecting FM associated with inflammatory rheumatic disease showed acceptable sensitivity (74.5%) and specificity (80.4%). The NPV was excellent (97%), whereas the PPV was poor (26%). High NPV and low PPV suggest that FiRST performs better for excluding the diagnosis of concomitant FM rather than confirming it [24].

For the FAST 4, a score of FAST4 ≥ 3 correctly classified 91.7% of patients compared to the 2011 FM criteria, with a sensitivity of 70.4% and a specificity of 97.1% [7]. We noticed that the specificity of the FAST 4 was higher than the FiRST score. In our study, we compared the FAST 4 to the FiRST score, which is routinely used in our practice. The advantage of calculating the FAST 4 index from the MDHAQ is that it allows for rapid screening using a single questionnaire, providing insights into the functional and psychological quality of life of our patients. Other indices developed from the MDHAQ also assess depression and anxiety [25,26]. To our knowledge, no study has compared the FAST 4 to the FiRST score.

FM can influence DAS28 scores due to increased pain sensitivity and symptom reporting, which may not correlate with actual inflammatory activity. Compared to other disease activity scores, DAS28 is particularly influenced by coexisting FM due to subjective components (tender joint count and VAS-global score) [22]. A prospective cross-sectional study including 270 RA patients found an association between the presence of FM and worse scores on the DAS28, HAQ, and SF-36 [20].

A study from the DANBIO (Danish Rheumatologic Database) registry, screening for concomitant FM performed by patient-administered questionnaires based on the modified 2010 ACR diagnostic criteria, revealed that the mean DAS28 in the FM group was 4.4 compared to 2.9 in the non-FM group (p < 0.001). They found that the elevated DAS28 in the FM group resulted from a high tender joint count (p = 0.003) and a high VAS-global score (p < 0.001). No significant differences were found in swollen joint counts or CRP levels [19]. A meta-analysis demonstrated an association between the presence of comorbid FM and worse DAS 28 with a mean DAS28 difference of 1.24 between RA with and without FM (95% CI: 1.10, 1.37) [5].

In contrast, a study on the impact of coexisting FM on disease activity in 126 patients with psoriatic arthritis (n = 64) and RA (n = 62) demonstrated that, unlike in psoriatic arthritis, no statistically significant correlation was observed between the FiRST score and disease activity parameters (CRP and DAS28) in RA patients [27].

FM may falsely increase the DAS28 score in RA, potentially biasing treatment decisions. Both overtreatment and undertreatment are possible. In our study, we did not find an association between CsDMARDs, bDMARDs, or corticosteroid use and patients with a positive FAST 4 Index. In contrast, PR Lage-Hansen et al. found more use of biological therapy in RA patients with concomitant FM [19]. Furthermore, a prospective cohort study with 256 RA patients found that RA patients with FM used more leflunomide and prednisone but had similar seven-year biologic-free survival [22]. Furthermore, much research demonstrated that the presence of fibromyalgia is one of the main predictors of not achieving remission following DMARD treatment [5].

To address the issue of concomitant FM and the risk of overtreating or undertreating FM patients, many studies suggest the use of ultrasound (US) evaluation of synovitis activity in RA patients [28,29]. In a real-life study of patients with RA and FM, a US examination was associated with less DMARD escalation and could reduce biological DMARD direct costs [30].

Our study has several strengths, including being among the first to investigate the prevalence of FM using the FAST 4 index. To the best of our knowledge, this is the first study to compare the FAST 4 and FiRST scores, providing valuable insights despite not being a validation or performance study. Nevertheless, our results represent a starting point for future research.

However, there are limitations to consider. First, the cross-sectional design limits our ability to establish causality for the observed associations. Second, the monocentric nature of the study restricts the generalization of our findings. Finally, because the study was not initially designed to compare the two scores, the results of this score comparison should be interpreted with caution.

## Conclusions

Our study suggests a high prevalence of concomitant FM in our population, highlighting the importance of screening for FM, particularly using the FAST 4 index based solely on the MDHAQ questionnaire. Additionally, the DAS28 score in RA patients with FM could misrepresent disease activity, potentially leading to mistreatment of this patient group.
